# The Great Cardiac Masquerade: Distinguishing Acute Coronary Syndrome From Myocarditis and Spontaneous Coronary Artery Dissection in a Middle-Aged Woman With Chest Pain

**DOI:** 10.7759/cureus.92219

**Published:** 2025-09-13

**Authors:** Lazaro Basart, Amina O Ali, Oscar Diaz, Amina A Mohamed, Alexandra Sirven, Fathimath Shifaly, Mariano Razzeto Rubio

**Affiliations:** 1 Internal Medicine, Palmetto General Hospital, Hialeah, USA; 2 Internal Medicine, American University of Antigua, St. John's, ATG; 3 Dr. Kiran C. Patel College of Osteopathic Medicine, Nova Southeastern University, Fort Lauderdale, USA

**Keywords:** : acute coronary syndrome, atypical spontaneous coronary artery dissection, cardiac cath, internal medicine in rural areas, underserved populations

## Abstract

Spontaneous coronary artery dissection (SCAD) is a rare, nonatherosclerotic cause of acute coronary syndrome (ACS), resulting from the formation of a false lumen and propagation of an intramural hematoma within the coronary artery wall. This process can lead to luminal narrowing, myocardial ischemia, and anginal symptoms. Because SCAD often presents with clinical and electrocardiographic features similar to obstructive ACS, as well as other close mimics such as myocarditis, misdiagnosis is common. Given the differences in management strategies, this can lead to suboptimal or even harmful outcomes. Here, we present the case of a 54-year-old woman with no traditional cardiovascular risk factors who presented with chest pain, had a nondiagnostic echocardiogram arguing against myocarditis, and was ultimately diagnosed with SCAD on coronary angiography.

## Introduction

Chest pain is among the most common reasons for emergency department visits and has a wide-ranging differential diagnosis, including cardiac, pulmonary, gastrointestinal, musculoskeletal, and psychogenic causes [1]. One of the most common cardiac causes of chest pain is acute coronary syndrome (ACS). ACS typically presents with classic symptoms such as crushing chest pain radiating to the left arm or jaw, and is more commonly reported in older men, though presentations can vary across age and sex. However, ACS can also manifest atypically, particularly in younger, healthier patients, leading to diagnostic uncertainty and potential delays in treatment [2]. Women, in particular, often fall outside the classic ACS demographic due to their age and lack of traditional risk factors such as diabetes mellitus or hypertension [3]. It is important to maintain a high index of suspicion when evaluating patients presenting with chest pain in the absence of other obvious causes and to ensure that common mimics do not obscure the diagnostic process.

ACS refers to a spectrum of conditions caused by atherosclerotic narrowing or rupture of the coronary arteries. Reduced perfusion to the myocardium leads to ischemia and anginal symptoms, and if left untreated, can progress to complete occlusion, resulting in myocardial infarction. While the hallmark presentation involves substernal chest pain with radiation to the left arm or jaw, typically brought on by exertion and relieved by nitroglycerin, this textbook description is not universal. In female patients lacking traditional risk factors, ACS may present with atypical symptoms such as epigastric discomfort, fatigue, generalized weakness, or even isolated nausea-often mimicking benign gastrointestinal or viral illnesses [2].

In addition to atypical presentation and demographic factors, the diagnostic process is further complicated by conditions that closely mimic ACS but arise from distinct mechanisms. Myocarditis, an inflammatory process often triggered by viral infection, autoimmune disease, or toxins, can present with chest pain, elevated cardiac enzymes, and ECG changes [4]. Similarly, spontaneous coronary artery dissection (SCAD) can present with near-identical findings and is a cause of non-atherosclerotic acute coronary syndrome [5]. Despite these overlaps, the underlying pathophysiology, treatment approach, and prognosis differ markedly from those of atherosclerotic ACS. 

The following case illustrates the diagnostic complexity in a 54-year-old woman with no traditional cardiovascular risk factors who presented to the emergency department with chest pain and was ultimately diagnosed with a SCAD.

## Case presentation

A 54-year-old Cuban woman with no past medical history presented to the emergency department of a community hospital that serves an underserved population due to an episode of chest pain that occurred in the morning at 8 a.m. while she was at rest in front of her computer. The chest pain had some typical features, such as its location in the center of her chest. She described it as an intermittent pressure-like sensation associated with generalized weakness, and it did not radiate. The patient denied shortness of breath, swelling in the lower extremities, and diaphoresis. She reported upper respiratory symptoms, including a runny nose and minimal coughing, that began five days prior.

Although hemodynamically stable, the patient’s blood pressure trended low, hovering in the 100s/50s. En route to the hospital, EMS administered three doses of nitroglycerin, which successfully resolved her symptoms. However, she experienced another episode of chest pain while in the ED and received another dose of nitroglycerin, which again alleviated the discomfort. The patient denied any cardiac history; notably, she did not have a history of hypertension. She denied any family history of cardiovascular disease before the age of 60.

**Figure 1:**
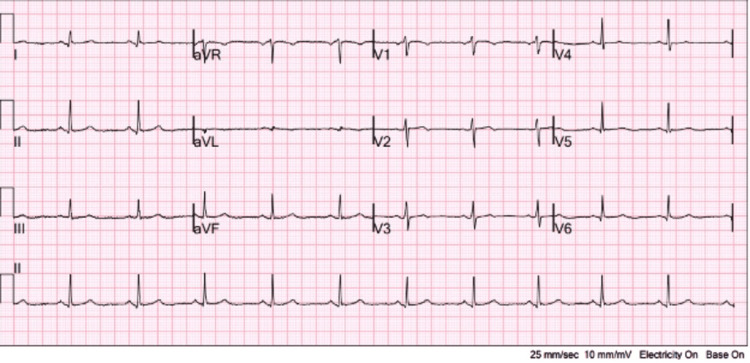
Initial EKG on admission showing nonspecific T-wave abnormalities

Although hemodynamically stable, the patient’s blood pressure trended low, hovering in the 100s/50s. On physical examination, she appeared comfortable and in no acute distress. She had no friction rub, dyspnea, jugular venous distention, peripheral edema, and her heart sounds were normal. An initial ECG performed in the ED on arrival showed no acute ischemic changes and nonspecific T-wave abnormalities, as shown in Figure 1. 

Transthoracic echocardiography revealed a preserved ejection fraction of 55-60% with mild concentric left ventricular hypertrophy. The left atrium was moderately dilated with slightly increased left atrial volume. There was thickening of the aortic valve leaflets with evidence of mild aortic stenosis. The mitral valve also demonstrated some calcification with mild mitral regurgitation. The ascending and descending aorta were of normal caliber and showed no evidence of significant atherosclerotic disease. The echo findings were suggestive of early cardiac remodeling, potentially secondary to an undiagnosed chronic hypertension. However, these findings did not explain the acute findings of chest pain and elevated cardiac enzymes in this patient.

**Figure 2:**
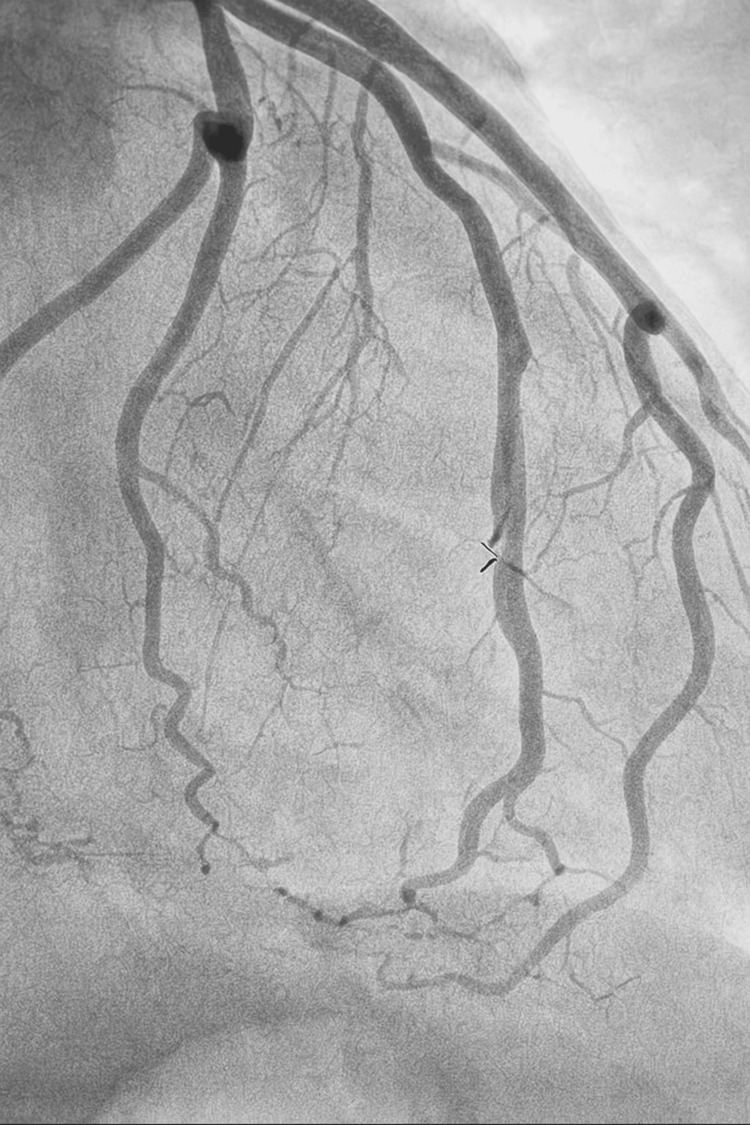
Cardiac catherization showing SCAD of the mid-distal ramus intermedius artery SCAD: spontaneous coronary artery dissection

Cardiac catheterization revealed a Type 2 SCAD of the mid-distal ramus intermedius artery, as shown in Figure 2. Despite the dissection, there was thrombolysis in myocardial infarction (TIMI)-3 flow distal to the lesion, indicating preserved myocardial perfusion. Given the adequate distal flow, the cardiology team recommended conservative management with close outpatient follow-up. The patient was discharged on dual antiplatelet therapy with aspirin and clopidogrel for one year, along with a statin and beta-blocker. Considering the absence of traditional cardiovascular risk factors, screening for fibromuscular dysplasia and connective tissue disorders was advised to evaluate for a potential underlying arteriopathy that may have contributed to the dissection. The patient was scheduled for outpatient cardiology follow-up, during which she will undergo regular ECGs, noninvasive imaging, and monitoring of disease progression and symptom control. Additionally, she will undergo outpatient testing for fibromuscular dysplasia and other connective tissue disorders.

## Discussion

Due to its low prevalence, SCAD is often underdiagnosed or misdiagnosed, particularly in young female patients presenting with chest pain [5]. In this case, the diagnostic process was further complicated by the patient’s recent viral prodrome, which raised concern for myocarditis. However, the patient's persistent chest pain necessitated a coronary angiography and saved time that would have been spent pursuing a myocarditis workup. Her absence of traditional cardiovascular risk factors further contributed to diagnostic uncertainty. As a relatively rare cause of acute coronary syndrome, SCAD is frequently overlooked during standard ACS evaluations, underscoring the need for heightened clinical suspicion in atypical presentations [6]. Early recognition is essential, as the management of SCAD depends on the type and severity of dissection and often differs significantly from that of other ACS mimics.

SCAD is a relatively rare cause of nonatherosclerotic ACS and can often be mistaken for atherosclerotic disease due to the similar angiographic appearance of coronary artery narrowing. However, SCAD results from a distinct mechanism: the formation of an intimal hematoma within the wall of the coronary artery, leading to luminal compression and myocardial ischemia [4]. Two primary hypotheses exist regarding its pathophysiology. One suggests that a tear in the intimal layer allows blood from the arterial lumen to enter the vessel wall, forming a false lumen [6]. The other posits that spontaneous hemorrhage from the vasa vasorum creates an intramural hematoma without any luminal disruption [5]. Most current research favors the latter, as angiographic studies often fail to show communication between true and false lumens [5]. Regardless of the initiating mechanism, the final outcome is critical luminal narrowing, which can lead to anginal symptoms and, in severe cases, myocardial infarction [7].

The typical SCAD patient is a middle-aged woman, often between the ages of 44 and 53, and frequently lacks traditional cardiovascular risk factors [5]. Approximately 90% of SCAD cases occur in women, suggesting a complex interplay of hormonal, genetic, and sex-related factors [5]. Although this high incidence points to a potential hormonal influence, particularly involving estrogen, the exact mechanisms remain unclear. SCAD accounts for approximately 1% of all myocardial infarctions, yet cohort studies indicate it may be responsible for up to one-third of MIs in women under 50 [7]. It is also implicated in 15-20% of myocardial infarctions occurring during pregnancy or the peripartum period [6]. Despite its relatively small contribution to overall MI incidence, SCAD has a disproportionately significant impact on young and pregnant women, and clinicians should maintain a high index of suspicion when evaluating chest pain in these populations [6]. 

The management of SCAD depends largely on the patient’s clinical stability and the angiographic type of dissection. SCAD is classified into four types based on its appearance on coronary angiography. Type 1 has the most classic appearance, with multiple radiolucent lumens or contrast staining of the arterial wall, clearly indicating a dissection [8]. Type 2 is more subtle, characterized by long, smooth luminal narrowing without visible signs of a tear or contrast leakage; this is the most common type [7]. Type 3 closely mimics atherosclerosis, appearing as focal or tubular stenosis, and often requires additional imaging for definitive diagnosis [9]. Type 4 represents complete vessel occlusion, which is difficult to distinguish from a thrombotic event at the time of angiography; the diagnosis is often made retrospectively after reperfusion is achieved [8].

Most patients presenting with SCAD are managed conservatively [10]. Only a small subset - those with more aggressive presentations or high-risk features - require invasive intervention. Studies have shown that over 90% of patients demonstrate spontaneous angiographic healing within one month of symptom onset [11]. Because of the potential for recurrence and symptom progression, long-term therapy with beta blockers and aspirin remains central to SCAD management [9]. Patients are typically hospitalized for 1-3 days for observation and discharged on medical therapy with close outpatient follow-up.

A minority of patients presenting with more severe symptoms - such as left main dissection, ongoing ischemia, ventricular arrhythmias, or signs of cardiogenic shock - may require urgent revascularization [9]. While percutaneous coronary intervention (PCI) is the mainstay of treatment in acute atherosclerotic coronary disease, it plays only a secondary and selective role in SCAD. PCI in SCAD carries lower success rates and higher complication risks due to vessel fragility, with a greater potential for iatrogenic dissection propagation [12]. As such, PCI is reserved for those with clear hemodynamic compromise or high-risk anatomical features. For hemodynamically stable patients with preserved distal flow, as in our case, current guidelines recommend medical therapy as first-line management [6].

Early recognition of SCAD is essential. Promptly ruling out other mimics not only helps guide appropriate therapy but also prevents patients from advancing deep into the wrong management algorithm - one that may expose them to unnecessary interventions and iatrogenic complications. Moreover, appreciating how the patient profile and pathophysiology of SCAD differ from those of atherosclerotic ACS is critical to avoiding delays in diagnosis and delivering optimal care.

## Conclusions

This report emphasizes the need to keep SCAD in mind when evaluating chest pain in otherwise healthy patients without traditional risk factors. The patient in this case presented with typical anginal symptoms that responded to nitroglycerin. Additional complexities in this case include the patient’s five-day viral prodrome and lack of traditional cardiovascular risk factors, which could have complicated the diagnostic process; fortunately, prompt angiography revealed the true culprit. The diagnosis of SCAD was ultimately confirmed, reinforcing its role as the gold standard in such cases.

Recognizing SCAD early is critical, as its treatment strategy diverges from that of atherosclerotic coronary disease. Patients with atherosclerotic ACS often require revascularization and stenting, whereas stable SCAD is typically managed conservatively. When coronary flow is preserved and no high-risk features are present, medical therapy with antiplatelets and beta blockers is generally sufficient. Invasive interventions such as PCI are reserved for severe or unstable cases, since they can increase the risk of worsening vessel injury in SCAD. Timely and accurate diagnosis ensures that patients receive appropriate management and are spared potentially harmful procedures.
